# Endosomal TLR-8 Senses microRNA-1294 Resulting in the Production of NFḱB Dependent Cytokines

**DOI:** 10.3389/fimmu.2019.02860

**Published:** 2019-12-06

**Authors:** Linda Pluta, Babak Yousefi, Blossom Damania, Asma A. Khan

**Affiliations:** ^1^Department of Microbiology and Immunology, School of Medicine, University of North Carolina at Chapel Hill, Chapel Hill, NC, United States; ^2^Department of Oral and Craniofacial Health Sciences, School of Dentistry, University of North Carolina at Chapel Hill, Chapel Hill, NC, United States; ^3^Department of Endodontics, Dental School, University of Texas Health Science Center at San Antonio, San Antonio, TX, United States

**Keywords:** microRNA, IL-8, cytokines, TLR-8, miR-1294, NF-κb

## Abstract

The primary function of toll-like receptor 8 (TLR-8) is the detection of viruses and other microbial pathogens. Recent evidence suggests that TLR-8 also senses host microRNAs (miRNAs) and implicate TLR-8 in autoimmune disorders. This study examined the interaction between miR-1294 and TLR-8. We first performed a BLAST search to identify miRNAs with the same sequences as two core motifs of miR-1294. Next, we examined NFḱB activation induced by the binding of miR-1294 mimic to endosomal TLR-8. HEK-Blue™ hTLR-8 cells (Invivogen), a HEK293 cell line co-transfected with human TLR-8 gene, were incubated with miR-1294 mimic. A TLR-8 agonist ssRNA40, was used as a positive control. Using the same experimental set up, we also examined the effects of miR-1294 and its two core motifs (Integrated DNA Technologies) on IL-8, IL-1β, and TNFα. Data were analyzed using *t*-test or one-way ANOVA and Dunnets *post-hoc* test. Using miRCarta we identified 29 other mature human miRNAs or their precursors which contain the same core motifs as miR-1294. Our data show that miR-1294 activates NFḱB in cells expressing TLR-8 (*p* < 0.05). miR-1294, and its core motifs induce expression of IL-8, IL-1β, and TNFα via TLR8 activation (*p* < 0.05). This constitutes a novel mechanism by which endosomal TLR-8 senses host miRNAs resulting in the release of pro-inflammatory cytokines and thus potentially contributing to autoimmune disorders.

## Introduction

Pathogen-associated molecular pattern receptors, including toll-like receptors (TLRs), are key molecules in the response to pathological stimuli such as bacterial endotoxins. TLRs are ubiquitously expressed by immune and non-immune cells including neurons, fibroblasts, endothelial cells, epithelial cells, and adipocytes. They recognize viral and microbial structures, and some endogenous molecules such as single stranded RNAs (1–3). Classic activation of TLRs depends upon the pathogen and the TLR expressing cell subtype (4).

To date, 11 human TLRs have been identified (5–8). Some TLRs, specifically TLRs 7, 8, and 9, recognize self molecules such as single stranded RNAs and play a role in autoimmunity. Recent studies show that TLR-7 recognizes microRNAs (miRNAs) resulting in the sensations of pain and itch (9). TLR-8 also recognizes miRNAs but differs from TLR-7 in multiple ways. TLR-8 is primarily expressed in monocytes, myeloid dendritic cells, and neutrophils while TLR-7 is expressed in B cells and plasmacytoid dendritic cells (10–12). TLR-8 is preferentially activated by ssRNA rich in AU sequences while TLR-7 is activated by sequences rich in GU (11). Moreover, unlike TLR-7, TLR-8 senses ssRNA through its ability to form secondary structures (13).

Nucleic acid-sensing TLRs (namely TLRs-7 and −8) are localized to endolysosomal compartments, where they encounter phagocytosed or endocytosed cargo (14). The subcellular localization of TLRs strategically favors the recognition of microbial (and not host) nucleic acids. Under normal conditions host nucleic acids are confined to other subcellular compartments and are not accessible to endosomal TLRs. Host nucleic acids in extracellular spaces are not taken up by endocytosis or phagocytosis. Instead they are rapidly degraded by nucleases unless they are stabilized by nucleic acid-binding proteins or are enclosed within extracellular vesicles (15, 16). In such conditions the host nucleic acids can reach endolysosomal compartments potentially leading to their recognition by TLRs and the development of autoimmunity.

The role of TLR-8 in autoimmune disease is yet to be fully explored. TLR-8 is implicated in the pathogenesis of several disorders including oral lichenoid reactions, irritable bowel syndrome (IBS), rheumatoid arthritis, systemic sclerosis and asthma (17–19). Blood levels of TLR-8 mRNA are higher in patients with systemic onset juvenile arthritis or Stills disease as compared to healthy controls (20). Furthermore, the levels of TLR-8 mRNA in these patients correlates positively with levels of IL-1β mRNA. Transgenic mice expressing high levels of human TLR-8 spontaneously develop arthritis while mice expressing low levels of TLR-8 do not (20). An *ex vivo* study incubated blood cells isolated from IBS patients with TLR agonists and assayed the supernatants for cytokine release (21). Cells from IBS patients responded to TLR-8 agonists in an exacerbated manner as compared to those from healthy controls. In a phase II clinical trial, administration of resiquimod, a TLR7/8 agonist, induced adverse events consistent with systemic cytokine induction at high doses (22). Studies on cancer show that tumor secreted miRNAs act as paracrine agonists of TLR-8 resulting in NFḱB activation and the release of pro-inflammatory cytokines (23). Thus, TLR-8 activation by host miRs may induce the release of proinflammatory cytokines.

We conducted a series of clinical studies on TLRs and miRNA expression profiles. Our studies on biopsies of inflamed human dental pulps show that on comparing pulps from patients experiencing moderate to severe tooth pain to those with no pain or mild pain, TLR-8 was the only differentially expressed TLR (24). In a separate study, we examined miRNA profiles in the blood from chronic pain patients. Specifically, these were patients with a single chronic pain condition-Vulvar Vestibulodynia alone (VBD alone) or patients with VBD and Irritable Bowel Syndrome (VBD + IBS) and compared them to pain-free controls (25). The miRNA profiles in the blood of chronic pain patients (VBD alone or VBD + IBS) differed from that of pain-free controls. We also noted an overlap in miRNA profiles between VBD alone and VBD + IBS. miR-1294 was differentially expressed in patients with VBD alone as well as in those with VBD + IBS. Another study reported results similar to our data with miR-1294, being differentially expressed in the blood of patients with chronic regional pain syndrome (26). To our knowledge, these are the only studies on miR-1294 in pain. Most studies on this miR are from the field of cancer research where it is reported to effect tumor progression (27–30).

Taken together these studies show that TLR-8 and miRNAs (including miR-1294) are differentially expressed in pain patients. The current study examined whether TLR-8 is activated by miR-1294 and its core motifs resulting in the production of pro-inflammatory cytokines.

## Materials and Methods

### Cell Culture

HEK-Blue™ hTLR-8 cells (Invivogen, San Diego, CA) are a stable commercial cell line that co-expresses TLR-8 gene and an optimized secreted embryonic alkaline phosphatase (SEAP) reporter gene into HEK293 cells. This SEAP reporter gene is controlled by IFN-β promotor fused to five NF-κB and AP-1 binding sites. With TLR-8 ligand stimulation, NF-κB and AP-1 are induced to produce SEAP.

Cells were grown according to manufactures' instruction in a maintenance media of Dulbecco's modified Eagle Media plus high glucose (4.5 g/l) and L-Glutamine (4 mM) (HyClone-GE Health Sciences, Logan, UT) supplemented with 10% v/v Fetal Bovine Serum (Gibco, Gaithersburg, MD), 50 U/ml Penicillin (Gibco), 50 μg/ml Streptomycin (Gibco), 100 μg/ml Normocin (Invivogen),30 μg/ml Blasticidin (Invivogen), and 100 μg/ml Zeocin (Invivogen) under standard conditions of 37°C, 5% CO_2_.

### NF-κB Activation

LyoVec complexed with miR-1294 was added to the HEK-Blue™ hTLR-8 cells. We initially used three different concentrations of miR1294 complexes- 200, 400, and 600 nM and incubated the cells for 18 and 24 h. As we got a robust response at 400 nM and 24 h, all subsequent experiments were conducted at that concentration and time point. Positive controls for the experiment were R848 (Resiquimod: an Imidazoquinoline compound, Invivogen), ssRNA40 (mimics viral ssRNA, Invivogen), and LyoVec transfection control (Invivogen). Negative controls included ssRNA41 (derived from ssRNA40 with replacement of U's by A's), LyoVec, hTLR-8 cells only and the parental line of hTLR-8 cells. Three biological replicate experiments plated on separate days were performed with individual samples placed in triplicate on each plate. Plates were analyzed spectrophotometrically at 620 nm wavelength on Spectra Max M2 (Molecular Devices, Sunnyvale, CA).

### Cytokine Gene and Protein Expression

We used the same experimental setup as described above to examine expression of cytokines at the transcript and protein levels. In addition to miR-1294 mimic, we also challenged the cells with two of its core motifs- Motif A-AUUGUUG and Motif B- AUUGUUA. To facilitate efficient gene expression analysis, we utilized the TaqMan^®^ Gene Expression Cells-to-CT™ Kit (Applied Biosystems/Thermo, Foster City, CA). Taqman primer assays were from Quant Studio 6 (Applied Biosystems/Thermo).

For analysis of protein expression, supernatants were collected from the plates after 24 h of incubation and placed on ice. Each sample was centrifuged (1,400 rpm for 1 min) to remove cellular debris and the supernatants placed in 1.5 ml micro-centrifuge tubes. Human Luminex (R&D systems, Minneapolis, MN) bead-based multiplex assays were used for the quantitation of the proteins. Multiplex assays were performed according to reagent manufacturer's instructions using a Bio-Plex 200 system (Bio-Rad, Hercules, CA) with Bio-Plex Manager v. 6.0 operating software. Samples along with the standards were performed in duplicate on multiple 96 well-format plates. All experiments were repeated at least three times.

### Identification of miRNAs With Similar Cores

We used the Basic Local alignment Search Tool (BLAST) to identify other microRNA with sequences similar to the core motifs AUUGUUG and AUUGUUA used in this study. The search was conducted on miRCarta, a central repository for miRNA candidates, and was restricted to human microRNAs.

### Statistical Analysis

Data were analyzed using GraphPad Prism software (La Jolla, CA, USA). Data is shown as mean ± SEM. Data were analyzed by one-way ANOVA and Dunnets *post-hoc* test. *P*-values <0.05 were considered significant.

## Results

### Several microRNAs Express the Same Core Motifs as miR-1294

*In silico* analysis identified 18 mature or precursor miRNAs which also contain the core motif AUUGUUG (Table 1) and seven mature or precursor miRNAs which contain the motif AUUGUUA (Table 2). Some of these have been reported to be expressed at higher levels in diseased tissue biopsies or in extracellular vesicles from patient populations. For example, miRNA-449a is expressed in high levels in breast cancer (31). Extracellular vesicles secreted by human carcinoma cell lines contain miR181d (32) and extracellular vesicles from patients with chronic regional pain syndrome contain >4-folds higher levels of miR-98 as compared to healthy controls (26). Deep sequencing of extracellular vesicles released by colon cancer cell lines shows that a subpopulation of them are enriched with both the miR-181 family as well as miR-98 (32). Given that extracellular vesicles are a form of intercellular communication, it is likely that the identified miRNAs can reach and activate endosomal TLR-8 to induce inflammation in target cells distant from the site of origin. Prior studies report that extracellular vesicular miRNAs released from cancer cells activate TLRs in immune cells (23). The target cells may also include neurons in the central nervous system as miRNAs packaged in extracellular vesicles are known to impact neuronal function. The functions of extracellular vesicular miRNAs are yet unclear. Further research is needed to understand how they modulate their target cells.

**Table 1:** *In silico* analysis identifying miRs that contain the core AUUGUUG.

**Name**	**Accession**	**miRCarta**	**Entity**
hsa-mir-181d	MI0003139	hsa-194-1489.1	Precursor
hsa-miR-181d-5p	MIMAT0002821	m-194	miRNA
hsa-mir-3065	MI0014228	hsa-454-247.1	Precursor
hsa-miR-3065-3p	MIMAT0015378	m-247	miRNA
hsa-mir-3145	MI0014170	hsa-1645-1465.1	Precursor
hsa-mir-3613	MI0016003	hsa-259-525.1	Precursor
hsa-mir-3655	MI0016055	hsa-2207.1	Precursor
hsa-mir-4774	MI0017417	hsa-2068-2326.1	Precursor
hsa-miR-601	MIMAT0003269	m-2255	miRNA
hsa-mir-601	MI0003614	hsa-2255.1	Precursor
hsa-mir-633	MI0003648	hsa-2111.1	Precursor
hsa-mir-6502	MI0022214	hsa-810-2463.1	precursor
hsa-miR-6502-5p	MIMAT0025460	m-810	miRNA
hsa-mir-6759	MI0022604	hsa-2074-2271.1	Precursor
hsa-mir-7849	MI0025519	hsa-1426.1	Precursor
hsa-miR-7849-3p	MIMAT0030424	m-1426	miRNA
hsa-mir-8066	MI0025902	hsa-2376.1	Precursor
hsa-mir-98	MI0000100	hsa-143-367.1	Precursor

**Table 2:** *In silico* analysis identifying miRs that contain the core AUUGUUA.

**Name**	**Accession**	**miRCarta**	**Entity**
hsa-mir-132	MI0000449	hsa-414-138.1	Precursor
hsa-miR-132-5p	MIMAT0004594	m-414	miRNA
hsa-miR-449a	MIMAT0001541	m-696	miRNA
hsa-mir-449a	MI0001648	hsa-696.1	precursor
hsa-mir-449b	MI0003673	hsa-923-1052.1	Precursor
hsa-miR-449b-5p	MIMAT0003327	m-923	miRNA
hsa-mir-569	MI0003576	hsa-2357.1	Precursor

### miR-1294 Induces NFκB Activation via TLR-8 Binding

**Figure 1:**
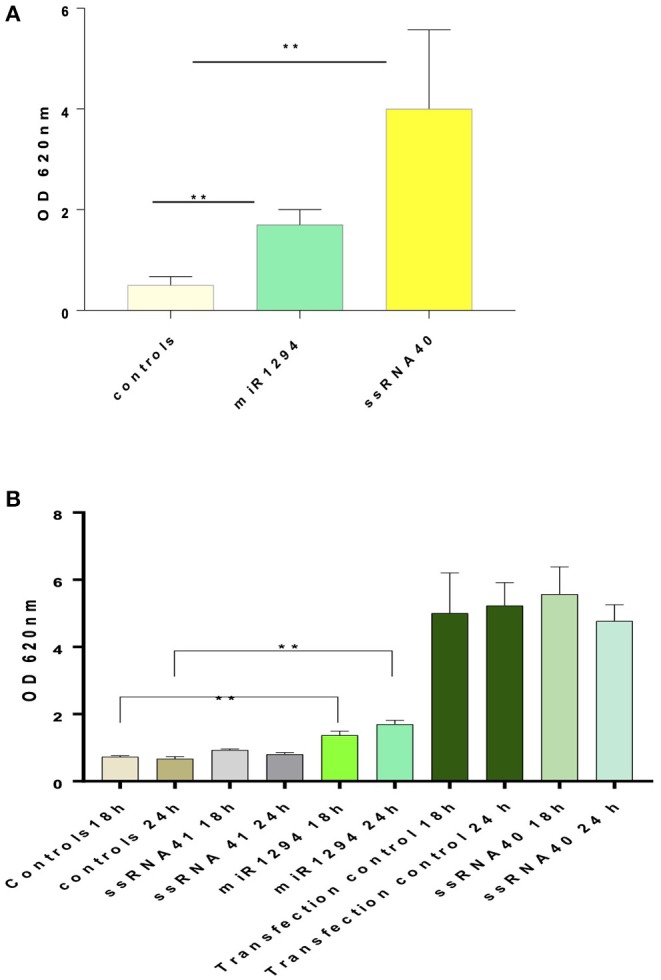
miR-1294 effects NFḱB-induced SEAP in TLR-8 expressing cells. **(A)** HEK-Blue™hTLR-8 cells (Invivogen, San Diego, CA), a stable commercial cell line co-transfected with hTLR-8 gene and an optimized secreted embryonic alkaline phosphatase (SEAP) reporter gene were incubated with miR-1294 mimic for 24 h. **(B)** HEK-Blue™hTLR-8 cells (Invivogen, San Diego, CA), were incubated with miR-1294 mimic or controls for 18 or 24 h. Negative controls were HEK-Blue™TLR8 cells alone and HEK-Blue™hTLR cells incubated with ssRNA4. Positive controls were HEK-Blue™hTLR8 cells incubated with ssRNA40 were and transfection controls. SEAP levels were quantified by spectrophotometry at 620 nm. Bars represent the mean of the three independent experiments with SEM. All experiments were replicated three times. Data analyzed by one-way ANOVA and Dunnet's *post-hoc* test. ^**^p < 0.01; ^***^p < 0.001.

Incubation of HEK-Blue™ hTLR-8 cells with miR-1294 induced robust production of NFκB-inducible secreted embryonic alkaline phosphatase (SEAP) (*p* < 0.01) (Figure 1A). Incubation with ssRNA40, a known TLR-8 agonist also induced SEAP production while ssRNA41 did not (Figure 1B). ssRNA41 is derived from ssRNA40 by replacement of all *U* nucleotides with adenosine and was used as a negative control.

### miR-1294 and Its Core Motifs Induce Cytokine Expression

**Figure 2:**
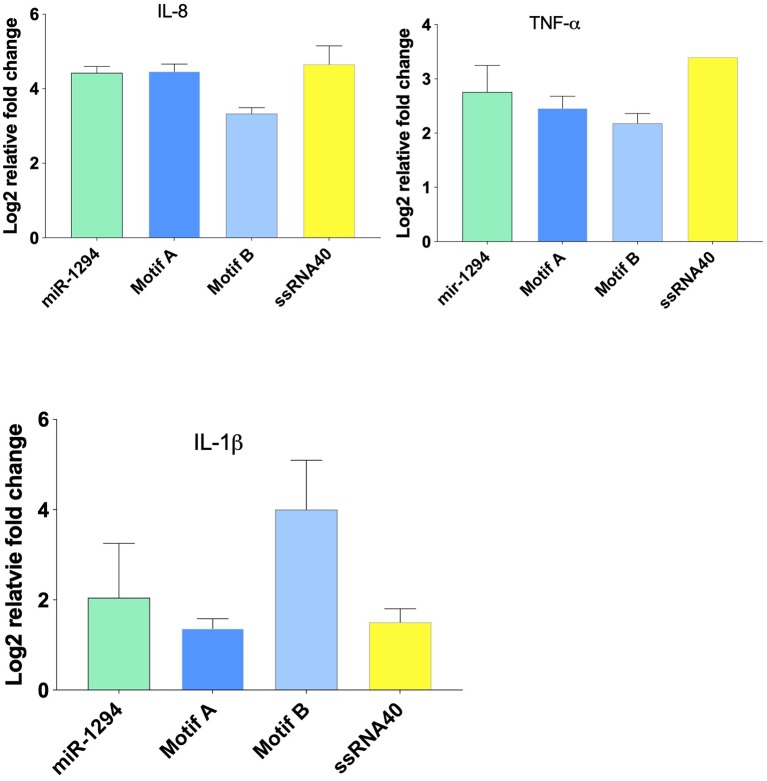
miR-1294 and its core motifs induce expression of IL-8, IL-1β, and TNFα mRNA. HEK-Blue™hTLR8 cells (Invivogen, San Diego, CA), were incubated with miR-1294 mimic and its two core motifs (Motif A-AUUGUUG and Motif B- AUUGUUA) for 24 h. ssRNA40, a TLR8 agonist, was used as a control. All experiments were replicated three times.

Incubation of HEK-Blue™ hTLR-8 cells with miR-1294 and its two core motifs induced significant increase in expression of IL-8, TNFα, and IL-1β mRNA (Figure 2). The effects of the two core motifs on IL-8 were not identical. Motif A induced a more robust expression of IL-8 mRNA and protein than motif B. This raises the possibility that the downstream signaling induced by these motifs differs.

**Figure 3:**
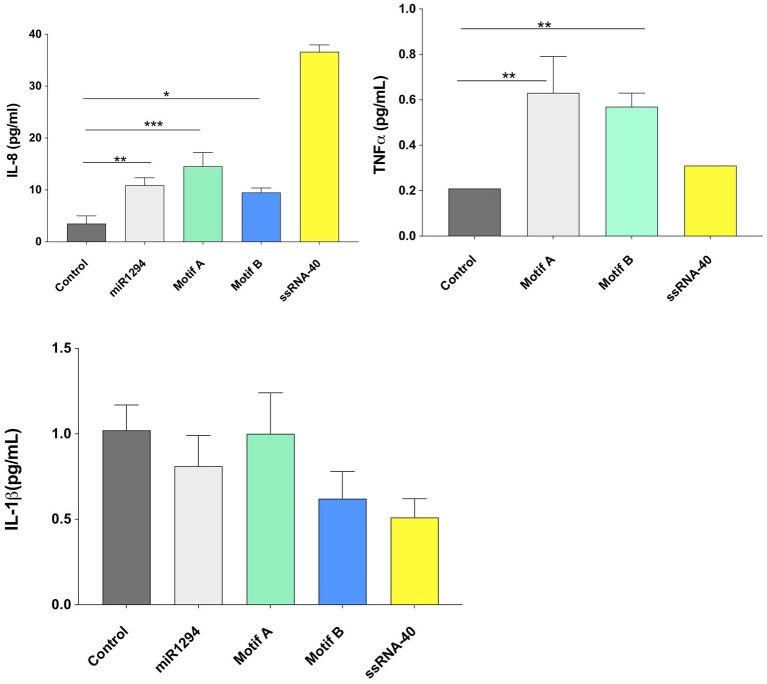
miR-1294 and its core motifs induce expression of IL-8 and TNFα but not IL-1β protein. HEK-Blue™hTLR8 cells (Invivogen, San Diego, CA), were incubated with miR-1294 mimic and its two core motifs (Motif A-AUUGUUG and Motif B- AUUGUUA) for 24 h. Supernatants were collected and assayed for levels of IL-8, TNFα, and IL-1 β. ssRNA40, a TLR8 agonist was used as a control. Data was analyzed by one-way ANOVA and Dunnet's *post-hoc* test. All experiments were replicated three times ^*^p < 0.05; ^**^p < 0.01; ^***^p < 0.001.

On examining cytokine protein expression in the supernatants, it was noted that miR-1294 and/or both of its core motifs induced significant increase in IL-8 and TNFα but not IL-1β (*p* < 0.05; Figure 3). The dissociation between IL-1β mRNA and protein synthesis has been reported in prior studies (33–35). The kinetics of IL-1 secretion are unique as compared to other secreted proteins. Large pools of precursor IL-1 accumulate intracellularly prior to release (35). Given that we assayed the supernatants, and not the cells, for IL-1β levels, it is likely that the cells themselves contained high levels of IL-1β or its precursor.

## Discussion

Altered expression of microRNAs is noted in several disorders including systemic sclerosis, inflammatory bowel disease, psoriatic arthritis, spinal muscular atrophy, and rheumatoid arthritis (21, 36, 37). miRNA expression profiles have been reported in effector cells as well as body fluids such as plasma and serum. In addition, altered miRNA expression has been documented in cancer, autoimmune disease, neurological disease, and cardiovascular disease. The distinct expression profiles of miRNAs in disease have raised the possibility that miRNA profiles may be used as biomarkers. However, the functional role of these miRNAs is only just being explored. Here we provide evidence that miR-1294 and its core motifs bind TLR-8 resulting in NFḱB activation and induction of pro-inflammatory cytokines.

Our initial studies on miR and TLR expression were conducted using biopsies of inflamed pulp tissues. Pulpitis represents an immune response to bacterial infection (38–40). Infections of the dental pulp typically involve a mixed, predominantly gram-negative and anaerobic bacterial flora and contain high levels of TNFα, IL-1, and IL-8 (41–44). Pulpal fibroblasts, immune cells, and neurons express TLRs, including TLR8, which recognize microbial components and activate NFκB, which in turn activates pro-inflammatory genes (40, 44). Pulpitis can be symptomatic with patients experiencing moderate to severe pain or it can be asymptomatic with no clinical symptoms of pain.

Using *in silico* analysis we identified several other miRNAs and their precursors which have the core motifs AUUGUUA and AUUGUUG. This raises the possibility that these miRNAs can bind and activate TLR-8 and induce cytokine synthesis. Some of the miRNAs that were identified are expressed at high levels in tissue biopsies and in exosomes. For example, miRNA-449a is expressed in high levels in breast cancer (31). Exosomes secreted by human carcinoma cell lines contain miR181d (32). Exosomes from patients with chronic regional pain syndrome contain >4-folds higher levels of miR-98 as compared to healthy controls (26). Given that exosomes are a form of intercellular communication, it is likely that the identified miRNAs can induce inflammation in target cells distant from the site of origin. Prior studies report that exosomal miRNAs released from cancer cells activate TLRs in immune cells (23). The target cells may also include neurons in the central nervous system as miRNAs packaged in exosomes are known to impact neuronal function. The function of exosomal miRNAs are yet unclear. Further research is needed to understand how they modulate their target cells.

IL-8 is a functionally diverse cytokine. It plays an important role in the immune response by inducing migratory and phagocytic activity and promoting angiogenesis. It is secreted by numerous cell types including endothelial cells, monocytes, neutrophils, and others (6). The synthesis and release of IL-8 is induced by various triggers including viral infections, bacterial lipopolysaccharides and cytokines such as IL-1, TNFα, Il-17, etc. Additionally, it can also inhibit the antiviral effects of interferon-α and thus enhance viral dissemination (45). Multiple lines of evidence support the role of IL-8 in pain hypersensitivity (46, 47). High levels of IL-8 are noted in diseases including chronic regional pain syndrome and pulpitis, both of which are associated with reports of intense pain (48, 49). To our knowledge this is the first report on activation of TLR-8 by host microRNA resulting in increased IL-8 expression.

In conclusion, our data show that miR-1294 and its two core motifs activate NFκB via TLR-8 resulting in increased expression of the cytokines IL-8, TNFα, and IL-1β. The core motifs examined here are also contained in other microRNAs. Thus, our findings have broader implications beyond miR-1294 and suggest that multiple miRNAs can potentially activate TLR-8 and induce cytokine synthesis. Given that aberrant miR expression is noted in multiple diseases, future research should be directed toward miR-TLR-8 interactions in various disease models.

## Data Availability Statement

The datasets generated for this study are available on request to the corresponding author.

## Author Contributions

AK contributed toward hypothesis generation, experiment design, data interpretation, and manuscript preparation. LP and BY conducted the experiments. BD contributed toward data interpretation.

### Conflict of Interest

The authors declare that the research was conducted in the absence of any commercial or financial relationships that could be construed as a potential conflict of interest.
